# Insertion mutants in *Drosophila melanogaster Hsc20* halt larval growth and lead to reduced iron–sulfur cluster enzyme activities and impaired iron homeostasis

**DOI:** 10.1007/s00775-013-0988-2

**Published:** 2013-02-27

**Authors:** Helge Uhrigshardt, Tracey A. Rouault, Fanis Missirlis

**Affiliations:** 1 Molecular Medicine Program, Eunice Kennedy Shriver National Institute of Child Health and Human Development, National Institutes of Health, Bethesda, MD 20892 USA; 2 Departamento de Fisiología, Biofísica y Neurociencias, Centro de Investigación y de Estudios Avanzados del Instituto Politécnico Nacional, CP 07360 Mexico City, Mexico

**Keywords:** Mitochondria, Iron–sulfur clusters, DnaJ protein, Iron regulatory protein, Iron regulatory element

## Abstract

Despite the prominence of iron–sulfur cluster (ISC) proteins in bioenergetics, intermediary metabolism, and redox regulation of cellular, mitochondrial, and nuclear processes, these proteins have been given scarce attention in *Drosophila*. Moreover, biosynthesis and delivery of ISCs to target proteins requires a highly regulated molecular network that spans different cellular compartments. The only *Drosophila* ISC biosynthetic protein studied to date is frataxin, in attempts to model Friedreich’s ataxia, a disease arising from reduced expression of the human frataxin homologue. One of several proteins involved in ISC biogenesis is heat shock protein cognate 20 (Hsc20). Here we characterize two *piggyBac* insertion mutants in *Drosophila Hsc20* that display larval growth arrest and deficiencies in aconitase and succinate dehydrogenase activities, but not in isocitrate dehydrogenase activity; phenotypes also observed with ubiquitous *frataxin* RNA interference. Furthermore, a disruption of iron homeostasis in the mutant flies was evidenced by an apparent reduction in induction of intestinal ferritin with ferric iron accumulating in a subcellular pattern reminiscent of mitochondria. These phenotypes were specific to intestinal cell types that regulate ferritin expression, but were notably absent in the iron cells where ferritin is constitutively expressed and apparently translated independently of iron regulatory protein 1A. *Hsc20* mutant flies represent an independent tool to disrupt ISC biogenesis in vivo without using the RNA interference machinery.

## Introduction

Iron–sulfur clusters (ISCs) may have participated in reactions leading to the origin of life on Earth [1, 2]. They are central to most key processes sustaining living ecosystems, including carbon and nitrogen fixation [3]. In animals, ISC proteins are involved in oxidative phosphorylation and the regulation of iron homeostasis [4, 5]. ISC proteins are also conspicuously abundant in the nucleus, but the specific functions for the ISCs in DNA replication and repair are currently under investigation [6–9]. Cells actively build and deliver ISCs to target proteins [10–13]; defects in this process lead to disease, notably Friedreich’s ataxia [14–16], which is caused by the expansion of a GAA trinucleotide repeat element in an intron of the human frataxin gene [17]. The study of related human disorders has revealed tissue-specific requirements of specific ISC biosynthesis genes [18–20].

One factor considered important for delivery of ISCs to target proteins, in particular under conditions of oxidative stress, is heat shock protein cognate 20 (Hsc20) [21, 22]. The yeast homologue of Hsc20 is known as Jac1 and was recovered from a genetic screen as a suppressor of superoxide dismutase deficiency [23]. Reduced activity of Jac1 resulted in a decrease in activity of iron/sulfur-containing mitochondrial proteins and an accumulation of iron in mitochondria [24–26]. Jac1 interacts with the iron–sulfur scaffold protein Isu1p [27] and this interaction appears to be conserved in evolution [21, 28]. Hsc20 proteins from higher animals contain a metal-binding, cysteine-rich N-terminal domain, which is important for the integrity and function of the human co-chaperone [21, 29].

ISC biosynthesis has been studied in *Drosophila*, following the cloning of *dfh*, the fly homologue of the human frataxin gene [30]. A number of laboratories have used RNA interference (RNAi) induced by the heterologous yeast Gal4/upstream activating sequence (UAS) transgenic induction system [31] to model Friedreich’s ataxia in *Drosophila* [32–36]. Strong ubiquitous reduction in *dfh* expression resulted in giant long-lived larvae that failed to initiate metamorphosis and had defects in ISC enzymes [32], whereas more moderate reductions in expression resulted in adult flies with locomotion defects and increased sensitivity to oxidative stress [33]. Scavenging of hydrogen peroxide but not of superoxide rescued many *dfh* phenotypes [34], including the reversal of inactivation of aconitase, whose ISC is a bona fide target for superoxide [37]. This result is consistent with a signaling role for hydrogen peroxide as recently uncovered in the fly hematopoietic and wound-healing processes [38–40]. Reduced *dfh* expression in the central nervous system led to defective mitochondrial axonal transport and membrane potential when RNAi was induced in neurons [35], and accumulation of lipids and lipid peroxidation when RNAi was induced in glia [36]. These studies and also observations that ubiquitous overexpression of *dfh* affected specifically the development of embryonic muscles [41] have highlighted the variable tissue-specific roles also for *Drosophila* *dfh*. As no animal models are available beyond *dfh* RNAi flies and frataxin knockout mice [42], we undertook to characterize two transgenic insertions disrupting the *Drosophila Hsc20* homologue.

## Materials and methods

### Drosophila stocks

All *Drosophila melanogaster* strains were maintained on standard cornmeal/yeast/agar medium at 25 °C. Iron supplementation was in the form of ferric ammonium citrate. *PBac{PB}l(3)72Do* ^*c05018*^ */TM6* and *PBac{WH}l(3)72Do* ^*f02457*^ */TM6*, *Tb* were from the Exelixis collection at Harvard Medical School. Both insertions were rebalanced to the *TM3*, *P{GAL4-Kr.C}DC2*, *P{UAS-GFP.S65T}DC10*, *Sb* ^*1*^ fluorescent balancer (GFP is green fluorescent protein) from Bloomington stock no. 5195 to identify the time of larval lethality and select homozygous mutant larvae for further analysis. The resulting stocks are abbreviated *Hsc20* ^*c5018*^ */TM3*, *Kr*-*GFP*, *Sb* and *Hsc20* ^*f2457*^ */TM3*, *Kr*-*GFP*, *Sb*, respectively, in the text. *Actin*-*Gal4* used for rescue experiments and *Fer1HCH* ^*G188*^ (Fer1HCH is ferritin-1 heavy chain homologue) used for visualization of intestinal ferritin have been described elsewhere [43].

### RNA isolation and reverse transcription PCR

**Fig. 1:**
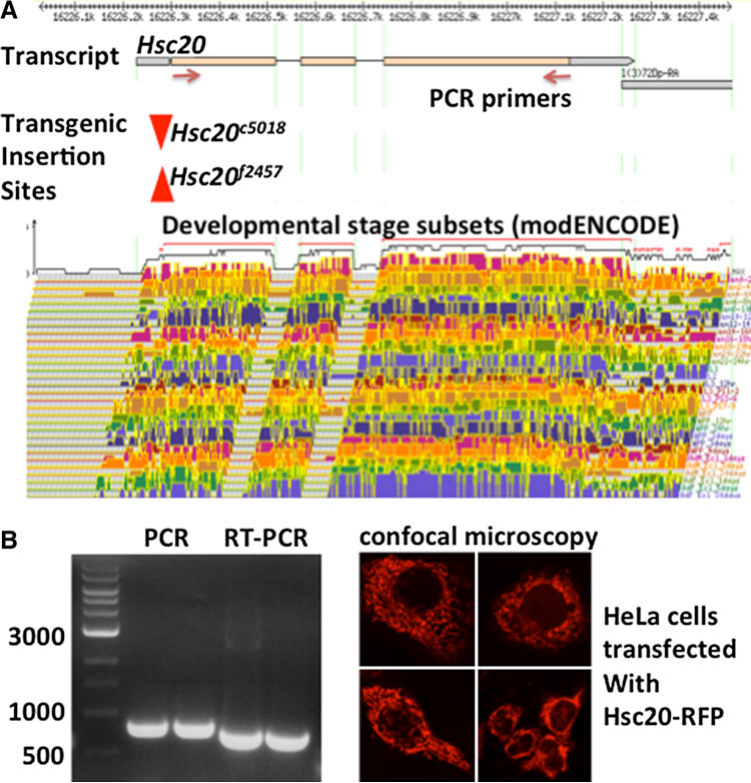
The *Drosophila Hsc20* gene is broadly expressed at low levels and encodes a mitochondrial protein. **a** The information presented was retrieved and adapted from the FlyBase and modENCODE databases, where *Hsc20* is listed as *l(3)72Do* or *CG34246*. The gene is composed of four exons and three introns. The open reading frame is shown in *beige* and the noncoding region of the messenger RNA in *gray*. The two *piggyBac* insertions used in this study disrupt the first exon. The gene shows ubiquitous low level of expression throughout development and into adulthood. **b** Confirmation of the presence and small size of introns 2 and 3 by reverse transcription PCR (*RT-PCR*; the location of primers used shown is in **a**) and localization of an Hsc20–red fluorescent protein (*RFP*) fusion protein in mitochondria of transfected HeLa cells

Total RNA was isolated from larvae using a modified Trizol method [44] and was purified using an RNeasy Mini kit (QIAGEN). The RNase-free DNase set (QIAGEN) was applied for on-column digestion of residual DNA. Total RNA of each sample was first reverse-transcribed into complementary DNA using the SuperScript III system (Invitrogen). The sequence encoding the full-length Hsc20 protein was amplified by PCR using the total match primers 5′-ATGAGTAGAGTTATTAATGGCTTTAAAAGTGCT-3′ and 5′-TCAGCTGCCCAGCAAACTTTGTTGCT-3′ (Fig. 1a). The PCR product was purified by agarose gel electrophoresis, excised, and eluted with a MinElute gel extraction kit (QIAGEN). Cloning of the PCR product was performed using a Zero Blunt TOPO PCR cloning kit (Invitrogen). For a C-terminal fusion of Hsc20 with red fluorescent protein, the sequence was amplified from the cloning vector with the primers hsc20NotF 5′-T*GAATTC*ATGAGTAGAGTTATTAATG GCTTTAAAGT-3′ and hsc20AgeR 5′-TT*ACCGGT*AATCCGCTGCCCAGCAAACT-3′. After subcloning in pCR2.1-TOPO, the transcript was excised with *Eco*RI and *Age*I and inserted in the corresponding sites of the mammalian expression vector pmCherry-N1 (Clontech). All constructs used were verified by sequencing.

### Cell culture and confocal microscopy

HeLa cells were obtained from the American Type Culture Collection and were grown in the Dulbecco’s modified Eagle’s medium with 4.5 g/l glucose, supplemented with 10 % (v/v) fetal bovine serum, 100 U/ml penicillin and 100 mg/ml streptomycin at 37 °C and 5 % CO_2_. Cells were seeded in microwell Lab-Tek chambered cover glass (Nunc International), and Fugene 6 (Roche) was used for transient transfections with mCherry-tagged Hsc20 according to the recommendations of the manufacturers. Immunofluorescence was imaged with a confocal microscope system (LSM 510 META; Zeiss) typically 24–36 h after transfection. For imaging red fluorescence, the 543-nm line of an He–Ne laser was used with a 488 nm/543 nm dichroic mirror, and the fluorescence was collected with a 560-nm long-pass filter.

### Enzyme assays

Three third instar larvae were transferred to a 1.5-ml microcentrifuge tube and were washed with 1× phosphate-buffered saline (PBS). Subsequently they were homogenized in about 5 vol of ice-cold lysis buffer [50 mM tris(hydroxymethyl)aminomethane (Tris)/HCl at pH 8.0, containing 1 % (v/v) NP-40 (Fluka), and EDTA-free protease inhibitor (one tablet per 10 ml) (Roche)] with a disposable plastic pestle and briefly sonicated on ice. The homogenates were cleared by centrifugation (16,000*g*, 4 °C, 15 min). Total aconitase activity was determined by following the reduction of NADP^+^ at 340 nm in the subsequent isocitrate dehydrogenase reaction. Five microliters of the homogenate was added to 50 μl buffer R1 [50 mM Tris/HCl, pH 8.0, with 50 mM NaCl, 5 mM MgCl_2_, 0.5 mM NADP^+^, 0.01 U isocitrate dehydrogenase (NADP^+^-dependent) from porcine heart (Sigma)]. The reaction was started by the addition of 45 μL buffer R2 (buffer R1 without NADP^+^ and isocitrate dehydrogenase, with 2.5 mM *cis*-aconitate). Activity was expressed as Δ*E*(340 nm)/Δ*t* and was normalized for protein concentration. Isocitrate dehydrogenase activity was determined by following the reduction of NADP^+^ at 340 nm as described above. Five microliters of lysate was added to 45 μl buffer R3 (buffer R1 without isocitrate dehydrogenase). The reaction was initiated by the addition of 50 μl buffer R4 (buffer R3 without NADP^+^, with 5 mM isocitrate).

### Succinate dehydrogenase activity in situ stain

Succinate dehydrogenase activity was evaluated on the basis of the succinate-dependent iodonitrotetrazolium chloride reduction. Prior to dissection the larvae were kept for 10 min in cold 1× PBS with 25 % (v/v) glycerol. Subsequently the cuticle and fat body of the larva (totally submerged in cold buffer) were carefully removed. The dissected tissue was briefly washed in cold isotonic buffer and was transferred on a coverslip. One hundred microliters of 50 mM Tris/HCl pH 7.5 with 1 mM rotenone, 1 mg/ml antimycin, 10 mM KCN, 25 mM azide, 0.1 % digitonin, and proteinase inhibitor was added to the sample. Tissues were completely submerged in this solution for 10 min. Following preincubation, 100 μl of 50 mM Tris/HCl pH 7.5 with 4 mM iodonitrotetrazolium chloride, 0.5 mM EDTA, 15 g/l CremophorEL (Sigma), and 50 mM succinate (for negative control without succinate) was added. Incubation was at room temperature for 10 min. The staining solution was removed with a pipette and filter paper and samples were imaged unfixed in 1× PBS with 25 % (v/v) glycerol. The reduction of iodonitrotetrazolium chloride leads to red, water-insoluble formazan.

### Enhanced Prussian blue stain

The dissection was performed as described in the previous section. The samples were rinsed three times with 1× PBS and subsequently fixed and stained simultaneously in 4 % paraformaldehyde (v/v) and Perls’s solution [1 % K_4_Fe(CN)_6_ and 1 % HCl] for 30 min at room temperature. Enhancement with diaminobenzidine was performed as described in [45].

## Results

### *DrosophilaHsc20* encodes a mitochondrial protein

An alignment between *Drosophila* and human Hsc20 protein sequences has revealed extended homology and 28 % identity between the homologue proteins of the two species [21]. The *Drosophila Hsc20* gene is currently annotated in FlyBase as *l(3)72Do*, following a detailed genetic analysis of the polytene chromosome region 72A-D that revealed *CG34246* as an essential gene [46]. Here we refer to *CG34246* or *l(3)72Do* as *Hsc20*.

Recent genomic approaches under the Model Organism Encyclopedia of DNA Elements (modENCODE) project [47], supported by the National Human Genome Research Institute, have provided a developmental transcriptome in *Drosophila melanogaster* [48]. This work has revealed that *Hsc20* contains four exons and is expressed at low levels throughout development (Fig. 1a) and in all tissues of larvae and adults (see http://flybase.org), consistent with a housekeeping role for a putative mitochondrial protein. The second intron is predicted to be 52 bp long and the third intron 60 bp long. Primers used to amplify the open reading frame of *Hsc20* by reverse transcription PCR confirmed the difference of 112 bp between the PCR on the genomic template and the reverse transcription PCR product and also that *Hsc20* is expressed in flies (Fig. 1b). A construct linking red fluorescent protein to the C-terminus of Hsc20 was transfected in HeLa cells to determine the subcellular localization of the protein, which was found to accumulate in mitochondria (Fig. 1c), a result that was expected on the basis of the characterization of the human protein and the conserved mitochondrial targeting sequence in the N-terminus of *Drosophila* Hsc20 [21].

### *DrosophilaHsc20* mutants show growth arrest and reduced ISC biogenesis

**Fig. 2:**
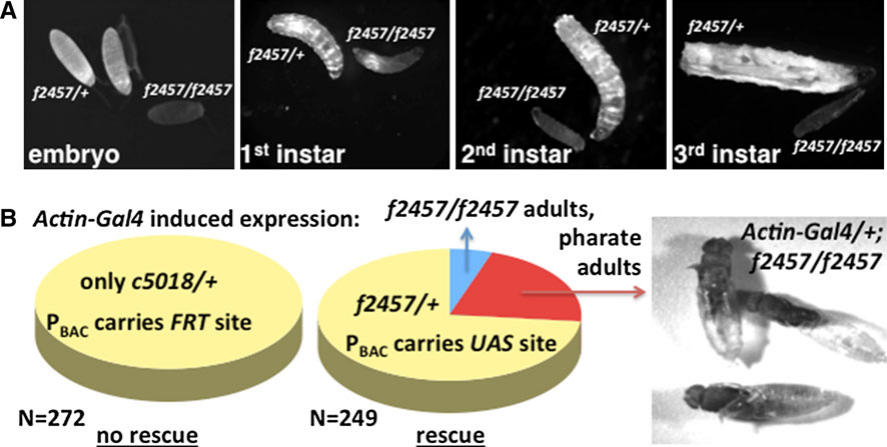
*Drosophila Hsc20* *piggyBac* insertion mutants arrest growth as third instar larvae. **a** *Hsc20* ^*f2457*^ */TM3*, *Kr*-*GFP*, *Sb* (*f2457/*+) flies (GFP is green fluorescent protein) laid eggs on agar plates supplied with yeast; embryos and larvae were visualized with a fluorescent microscope and homozygous mutants (*f2457/f2457*) were identifiable from the absence of fluorescent signal. Homozygous mutant larvae failed to grow during the third instar. **b** *Actin*-*Gal4*-driven genetic rescue of homozygous *f2457/f2457* mutants by virtue of the upstream activating sequence (*UAS*) elements present in the *PBac{WH}l(3)72Do* ^*f02457*^ transgene, which permit expression of the endogenous *Hsc20* gene following activation by Gal4. *N* depicts the total number of flies scored. The rescue is not always complete, as many flies complete metamorphosis but fail to eclose from their pupal cases. UAS elements are absent from the *PBac{PB}l(3)72Do* ^*c05018*^ transgene, which serves as a control for this experiment. *FRT* flippase recombinase target

The two transgenic insertions used in this study were selected because their insertion points disrupt the first exon of *Hsc20* at a position in the 5′ untranslated region (Fig. 1a). Consistent with what was shown previously for other transposons in this locus [46], both the *Hsc20* ^*c5018*^ allele and the *Hsc20* ^*f2457*^ allele are homozygous lethal and do not complement each other. The developmental time point of lethality was investigated by balancing the respective insertions to *TM3*, *Kr*-*GFP*, *Sb*, making heterozygous and homozygous genotypes recognizable through the presence or absence of fluorescence, respectively, throughout the organism’s development (Fig. 2a). The time point of lethality was identical for both alleles (shown only for the *Hsc20* ^*f2457/f2457*^ genotype); homozygous mutant larvae grew through the first and second instars, but halted growth after they reached the third instar (Fig. 2a).

As the *piggyBac* element inserted in the *Hsc20* ^*f2457*^ allele included UAS, which can be used to induce neighboring gene expression (from exons 2-4 that encode the Hsc20 open reading frame), we generated *Actin*-*Gal4/Cyo; Hsc20* ^*f2457*^ */TM3*, *Kr*-*GFP*, *Sb* flies and looked for rescue (Fig. 2b). Given that balancer chromosomes are homozygous lethal, a full rescue would result in 33 % *Hsc20* ^*f2457/f2457*^ homozygous adult flies and 67 % *Hsc20* ^*f2457/*+^ heterozygous adult flies. In the experiment shown we counted 249 progeny flies, of which 183 (73 %) were heterozygous and 66 (27 %) were homozygous, of which 53 (22 %) died while trying to eclose from their pupal cases (Fig. 2b, left panel) and only 13 (5 %) survived into adulthood. The *Actin*-*Gal4/Cyo; Hsc20* ^*c5018*^ */TM3*, *Kr*-*GFP*, *Sb* genotype was also generated as a control, but no homozygous viable adults were obtained in this stock at any point. Hence, our rescue experiments show that the lethality associated with *Hsc20* ^*f2457/f2457*^ flies is due to a specific reduction in *Hsc20* expression.

**Fig. 3:**
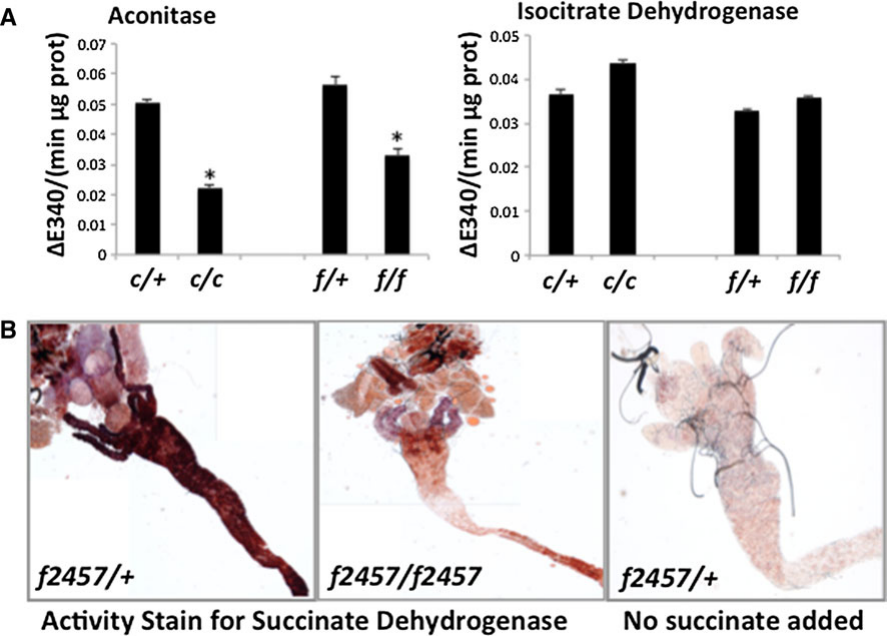
*Drosophila Hsc20* mutants have reduced aconitase and succinate dehydrogenase activities but normal isocitrate dehydrogenase activity, suggesting specific defects in iron–sulfur cluster (ISC) biosynthesis. **a** Lysates were prepared from heterozygous *Hsc20* ^*c5018*^ */TM3*, *Kr*-*GFP*, *Sb* (*c/+*) and *Hsc20* ^*f2457*^ */TM3*, *Kr*-*GFP*, *Sb* (*f/+*) adult files and homozygous *Hsc20* ^*c5018*^/*Hsc20* ^*c5018*^ (*c/c*) and *Hsc20* ^*f2457*^ */Hsc20* ^*f2457*^ (*f/f*) adult flies. Lysates were assayed for ISC-dependent aconitase activity and for ISC-independent isocitrate dehydrogenase activity and normalized against total protein. Note the differences in *Hsc20* homozygous mutants for aconitase (*asterisks* denote *p* < 0.01 from the *t* test). **b** Succinate dehydrogenase activity normally present in the anterior midgut is severely reduced in intestines from *Hsc20* ^*f2457*^ */Hsc20* ^*f2457*^ larvae. Specificity of this stain was shown by removal of the substrate for the reaction in the *left panel*, in which case minimal signal is obtained

We collected third instar homozygous *Hsc20* mutant larvae and their heterozygous sibling controls and quantified total aconitase and isocitrate dehydrogenase activities in lysates prepared from these. Both *Hsc20* ^*c5018/c5018*^ and *Hsc20* ^*f2457/f2457*^ larvae showed a marked decrease of aconitase activity to approximately 50 % of the activity measured in the respective heterozygous flies (Fig. 3a, left panel). To verify that the loss of activity was due to a specific loss of ISCs in aconitase enzymes, we also tested isocitrate dehydrogenase, another housekeeping enzyme that does not depend on ISCs, in the same lysates and found no difference between the genotypes (Fig. 3a, right panel). We also tested for succinate dehydrogenase activity, this time employing an activity assay in dissected tissues of the larvae. We observed high activity in the anterior midgut of the intestine in *Hsc20* ^*f2457/*+^ larvae, which was markedly reduced in *Hsc20* ^*f2457/f2457*^ larvae (Fig. 3b). These results showed that ISC proteins are specifically impaired in *Hsc20* mutant larvae.

### *DrosophilaHsc20* mutants accumulate iron in mitochondria instead of in ferritin

**Fig. 4:**
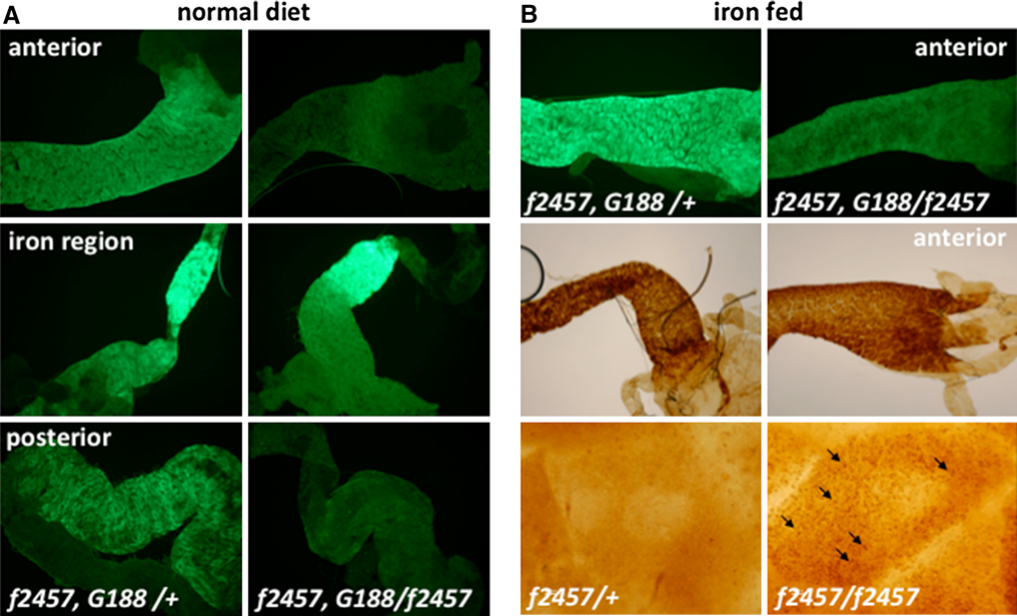
*Drosophila Hsc20* mutants show punctate iron accumulation and fail to induce ferritin in the intestine. **a** A GFP–ferritin protein trap line [43] was crossed into *Hsc20* mutants to monitor ferritin accumulation. Intestines were dissected from *Hsc20* ^*f2457*^, *Fer1HCH* ^*G188*^ */TM3*, *Sb* (*f2457*, *G188/*+) heterozygous control flies (Fer1HCH is ferritin-1 heavy chain homologue) and *Hsc20* ^*f2457*^, *Fer1HCH* ^*G188*^ */Hsc20* ^*f2457*^ mutants (*f2457*, *G188/f2457*). Note that induction of ferritin appears to be reduced in the anterior and posterior midgut, but is unaffected in the iron region of the middle midgut. **b** Feeding larvae with iron normally leads to a pronounced induction of ferritin in the anterior midgut, not seen in *Hsc20* mutants, which still accumulate iron in the anterior midgut, but in a pattern more reminiscent of mitochondria (*arrows*)

To test the effect of defective ISC biogenesis on iron homeostasis, we recombined a GFP–ferritin trap line [43, 49] with the *piggyBac* insertions in *Hsc20*. We then dissected intestines from *Hsc20* ^*f2457*^, *Fer1HCH* ^*G188*^ */Hsc20* ^*f2457*^ third instar larvae and from *Hsc20* ^*f2457*^, *Fer1HCH* ^*G188*^ */TM3*, *Sb* controls and observed ferritin under a fluorescence microscope. Ferritin accumulation in the iron region was unaffected between the two genotypes (Fig. 4a, middle panels); expression of ferritin in these cells was previously shown to be independent of iron [49]. In contrast, cells in the anterior and posterior midgut showed low, but readily detectable levels of ferritin accumulation in the heterozygous *Hsc20* mutant larvae; these cells were devoid of ferritin in the homozygous *Hsc20* mutants (Fig. 4a, top and bottom panels). Dietary iron induces ferritin in the anterior midgut of larvae [43, 49], so we tested the response of *Hsc20* mutants when grown on yeast supplemented with 1 mM ferric ammonium citrate. As expected, *Hsc20* ^*f2457*^, *Fer1HCH* ^*G188*^ */TM3*, *Sb* larvae showed a clear induction of ferritin in the anterior midgut, whereas induction of ferritin in *Hsc20* ^*f2457*^, *Fer1HCH* ^*G188*^ */Hsc20* ^*f2457*^ intestines was compromised (Fig. 4b, top panels).

Ferritin induction in the anterior midgut serves as a store for iron absorbed by the diet [43]. We stained the intestines dissected from *Hsc20* ^*f2457/f2457*^ larvae (and from the respective controls) for iron using the enhanced Prussian blue stain (Fig. 4b, middle panels). Despite the absence of ferritin induction in *Hsc20* ^*f2457/f2457*^ mutants, we still observed marked accumulation of iron in the anterior midgut. Examination of the stained cells at higher magnification revealed a punctate pattern, which was not seen in *Hsc20* ^*f2457/*+^ heterozygous control stains (Fig. 4b, bottom panels).

## Discussion

### *Hsc20* encodes a mitochondrial protein involved in ISC biogenesis

We have characterized two *piggyBac* insertions in the *Hsc20* gene that result in reduced activities of classic ISC enzymatic activities and have shown that Hsc20 is a nuclear-encoded mitochondrial protein. Our results are largely similar to those obtained by other investigators who studied ubiquitous RNAi phenotypes of the *dfh* gene [32, 33] and therefore validate our conclusion that the *Hsc20* mutants described here provide the first example of *Drosophila* mutants in ISC biogenesis.

### Iron accumulation in mitochondria is characteristic of disrupted ISC biosynthesis

We observed a punctate staining for iron in *Hsc20* mutant cells localized in the anterior intestine of larvae (Fig. 4b, lower panels). Accumulation of iron in mitochondria is a characteristic of Friedreich’s ataxia patients and is also observed in animal models of the disease [50] and yeast lacking Jac1 [24, 26]. Such pathologic iron is present in aggregate form, but is clearly distinct from ferritin iron [51]. Given the evidence accumulated in studies with yeast, mice models, and human patients [42], we believe that the punctate staining we have observed specifically in *Hsc20* mutants could represent similar mitochondrial iron inclusions, suggesting these may also develop in the new *Drosophila* model of disrupted ISC biogenesis we have described. The phenotypes of reduced ISC activities, mitochondrial iron overload, and cytosolic iron deficiency are consistent with those reported for the human and yeast Hsc20 homologues [21–29]. For these reasons we suggest that *Hsc20* should be considered as a candidate disease gene in humans.

### Cell-type-specific effects in ferritin regulation in *Hsc20* mutants

Intestinal ferritin regulation is complex and regulates both iron absorption and iron storage [43, 52–54]. The iron cells in the middle midgut express ferritin in a constitutive manner and independent of systemic iron concentrations [49]. Cells in the very posterior of the intestine appear to be specialized in zinc homeostasis, where ferritin may also have a specialized function that is not yet understood [55]. In this study, we have noted no changes in ferritin accumulation in the middle midgut in *Hsc20* mutants (Fig. 4a, middle panels), consistent with the notion that iron homeostasis in these cells is specialized. The apparent downregulation of ferritin in cells of the anterior midgut (Fig. 4a, b, upper panels) may be explained by the activation of iron regulatory protein 1A as a result of defective ISC biogenesis and hence translational repression of ferritin [56, 57], or by mitochondrial iron accumulation causing a relative cytoplasmic iron deficiency. Whichever explanation holds (or both may be true), we note that not all cell types respond in the same way on genetic inactivation of *Hsc20*, calling for further investigations into the in vivo roles of the ISC biosynthetic machinery. The mutants described in this study should help expand in vivo studies.

### An iron-related mitochondrial to nucleus signal may be relevant to the circadian clock

*Drosophila melanogaster* provides an elegant system for the parallel investigation of molecular, biochemical, cellular, physiological, and behavioral biology using genetic and environmental manipulations. The *Hsc20* mutants we describe offer an alternative model to validate results obtained via RNAi experiments [32–36, 58]. Most other genes known from other systems to be involved in ISC biosynthesis are conserved in *Drosophila*, where they were implicated in the maintenance of circadian rhythms [58], representing the first proposed function of ISCs in the regulation of animal behavior. At least one ISC-containing enzyme, dihydropyrimidine dehydrogenase, has been shown to have circadian rhythmic expression in the head of flies [59], but it is unknown which ISC protein may interact directly with the biological clock’s transcriptional feedback loops [60, 61]. The function of ISC proteins in regulating redox homeostatic mechanisms may help explain how the recently discovered free-running metabolic and redox cycles link to the cellular transcriptional and translational timekeeping networks [62, 63]. In plants, a retrograde signal from chloroplasts to nucleus signaling an iron deficiency has been suggested to affect the period length of the clock [64–66]. Such a signal could be the unknown chemical exported by the ABCB7 mitochondrial transporter [17, 67]. Owing to the lethality of *Hsc20* mutants during their larval stage of development (Fig. 2a), we were unable to test whether loss of Hsc20 would disrupt the circadian clock. However, this lethality underscores the essential role of ISCs in animal growth.

## Abbreviations

Fer1HCH: Ferritin-1 heavy chain homologue
GFP: Green fluorescent protein
Hsc20: Heat shock protein cognate 20
ISC: Iron–sulfur cluster
modENCODE: Model Organism Encyclopedia of DNA Elements
PBS: Phosphate-buffered saline
RNAi: RNA interference
Tris: Tris(hydroxymethyl)aminomethane
UAS: Upstream activating sequence

## References

[1] Wickramasinghe RH (1973). Space Life Sci.

[2] Schoepp-Cothenet B, van Lis R, Philippot P, Magalon A, Russell MJ, Nitschke W (2012). Sci Rep.

[3] Hall DO, Evans MC (1969). Nature.

[4] Crack JC, Green J, Thomson AJ, Le Brun NE (2012). Curr Opin Chem Biol.

[5] Tong WH, Rouault TA (2007). Biometals.

[6] Stehling O, Vashisht AA, Mascarenhas J, Jonsson ZO, Sharma T, Netz DJ, Pierik AJ, Wohlschlegel JA, Lill R (2012). Science.

[7] Gari K, Leon Ortiz AM, Borel V, Flynn H, Skehel JM, Boulton SJ (2012). Science.

[8] Benjdia A (2012). Curr Opin Struct Biol.

[9] White MF, Dillingham MS (2012). Curr Opin Struct Biol.

[10] Rouault TA (2012). Dis Model Mech.

[11] Rouault TA, Tong WH (2005). Nat Rev Mol Cell Biol.

[12] Shepard EM, Boyd ES, Broderick JB, Peters JW (2011). Curr Opin Chem Biol.

[13] Lill R, Hoffmann B, Molik S, Pierik AJ, Rietzschel N, Stehling O, Uzarska MA, Webert H, Wilbrecht C, Muhlenhoff U (2012). Biochim Biophys Acta.

[14] Rouault TA, Tong WH (2008). Trends Genet.

[15] Pandolfo M, Pastore A (2009). J Neurol.

[16] Stemmler TL, Lesuisse E, Pain D, Dancis A (2010). J Biol Chem.

[17] Campuzano V, Montermini L, Moltò MD, Pianese L, Cossée M, Cavalcanti F, Monros E, Rodius F, Duclos F, Monticelli A, Zara F, Cañizares J, Koutnikova H, Bidichandani SI, Gellera C, Brice A, Trouillas P, De Michele G, Filla A, De Frutos R, Palau F, Patel PI, Di Donato S, Mandel JL, Cocozza S, Koenig M, Pandolfo M (1996). Science.

[18] Sanaker PS, Toompuu M, Hogan VE, He L, Tzoulis C, Chrzanowska-Lightowlers ZM, Taylor RW, Bindoff LA (2010). Biochim Biophys Acta.

[19] Crooks DR, Jeong SY, Tong WH, Ghosh MC, Olivierre H, Haller RG, Rouault TA (2012). J Biol Chem.

[20] Xia H, Cao Y, Dai X, Marelja Z, Zhou D, Mo R, Al-Mahdawi S, Pook MA, Leimkuhler S, Rouault TA, Li K (2012). PLoS ONE.

[21] Uhrigshardt H, Singh A, Kovtunovych G, Ghosh M, Rouault TA (2010). Hum Mol Genet.

[22] Shan Y, Cortopassi G (2012). Hum Mol Genet.

[23] Strain J, Lorenz CR, Bode J, Garland S, Smolen GA, Ta DT, Vickery LE, Culotta VC (1998). J Biol Chem.

[24] Voisine C, Cheng YC, Ohlson M, Schilke B, Hoff K, Beinert H, Marszalek J, Craig EA (2001). Proc Natl Acad Sci USA.

[25] Lutz T, Westermann B, Neupert W, Herrmann JM (2001). J Mol Biol.

[26] Kim R, Saxena S, Gordon DM, Pain D, Dancis A (2001). J Biol Chem.

[27] Andrew AJ, Dutkiewicz R, Knieszner H, Craig EA, Marszalek J (2006). J Biol Chem.

[28] Ciesielski SJ, Schilke BA, Osipiuk J, Bigelow L, Mulligan R, Majewska J, Joachimiak A, Marszalek J, Craig EA, Dutkiewicz R (2012). J Mol Biol.

[29] Bitto E, Bingman CA, Bittova L, Kondrashov DA, Bannen RM, Fox BG, Markley JL, Phillips GN (2008). J Biol Chem.

[30] Canizares J, Blanca JM, Navarro JA, Monros E, Palau F, Molto MD (2000). Gene.

[31] Martinek S, Young MW (2000). Genetics.

[32] Anderson PR, Kirby K, Hilliker AJ, Phillips JP (2005). Hum Mol Genet.

[33] Llorens JV, Navarro JA, Martinez-Sebastian MJ, Baylies MK, Schneuwly S, Botella JA, Molto MD (2007). FASEB J.

[34] Anderson PR, Kirby K, Orr WC, Hilliker AJ, Phillips JP (2008). Proc Natl Acad Sci USA.

[35] Shidara Y, Hollenbeck PJ (2010). J Neurosci.

[36] Navarro JA, Ohmann E, Sanchez D, Botella JA, Liebisch G, Molto MD, Ganfornina MD, Schmitz G, Schneuwly S (2010). Hum Mol Genet.

[37] Missirlis F, Hu J, Kirby K, Hilliker AJ, Rouault TA, Phillips JP (2003). J Biol Chem.

[38] Owusu-Ansah E, Banerjee U (2009). Nature.

[39] Moreira S, Stramer B, Evans I, Wood W, Martin P (2010). Curr Biol.

[40] Juarez MT, Patterson RA, Sandoval-Guillen E, McGinnis W (2011). PLoS Genet.

[41] Navarro JA, Llorens JV, Soriano S, Botella JA, Schneuwly S, Martinez-Sebastian MJ, Molto MD (2011). PLoS ONE.

[42] Martelli A, Napierala M, Puccio H (2012). Dis Model Mech.

[43] Missirlis F, Kosmidis S, Brody T, Mavrakis M, Holmberg S, Odenwald WF, Skoulakis EM, Rouault TA (2007). Genetics.

[44] Bogart K, Andrews J (2006). CGB technical report 2006–2010.

[45] Tong WH, Rouault TA (2006). Cell Metab.

[46] Cooper MT, Kennison JA (2011). PLoS ONE.

[47] modENCODE Consortium (2010) Science 330:1787–1797 10.1126/science.1198374 PMC3192495 21177974

[48] Graveley BR, Brooks AN, Carlson JW, Duff MO, Landolin JM, Yang L, Artieri CG, van Baren MJ, Boley N, Booth BW, Brown JB, Cherbas L, Davis CA, Dobin A, Li R, Lin W, Malone JH, Mattiuzzo NR, Miller D, Sturgill D, Tuch BB, Zaleski C, Zhang D, Blanchette M, Dudoit S, Eads B, Green RE, Hammonds A, Jiang L, Kapranov P, Langton L, Perrimon N, Sandler JE, Wan KH, Willingham A, Zhang Y, Zou Y, Andrews J, Bickel PJ, Brenner SE, Brent MR, Cherbas P, Gingeras TR, Hoskins RA, Kaufman TC, Oliver B, Celniker SE (2011). Nature.

[49] Mehta A, Deshpande A, Bettedi L, Missirlis F (2009). Biochimie.

[50] Puccio H, Simon D, Cossee M, Criqui-Filipe P, Tiziano F, Melki J, Hindelang C, Matyas R, Rustin P, Koenig M (2001). Nat Genet.

[51] Whitnall M, Rahmanto YS, Huang ML, Saletta F, Lok HC, Gutierrez L, Lazaro FJ, Fleming AJ, St Pierre TG, Mikhael MR, Ponka P, Richardson DR (2012) Proc Natl Acad Sci USA. doi:10.1073/pnas.1215349109 10.1073/pnas.1215349109 PMC3528580 23169664

[52] Georgieva T, Dunkov BC, Harizanova N, Ralchev K, Law JH (1999). Proc Natl Acad Sci USA.

[53] Tang X, Zhou B (2012). FASEB J.

[54] Kosmidis S, Botella JA, Mandilaras K, Schneuwly S, Skoulakis EM, Rouault TA, Missirlis F (2011). Neurobiol Dis.

[55] Gutierrez L, Sabaratnam N, Aktar R, Bettedi L, Mandilaras K, Missirlis F (2010). FEBS Lett.

[56] Lind MI, Missirlis F, Melefors O, Uhrigshardt H, Kirby K, Phillips JP, Soderhall K, Rouault TA (2006). J Biol Chem.

[57] Surdej P, Richman L, Kuhn LC (2008). Insect Biochem Mol Biol.

[58] Mandilaras K, Missirlis F (2012). Metallomics.

[59] Van Gelder RN, Bae H, Palazzolo MJ, Krasnow MA (1995). Curr Biol.

[60] Veleri S, Brandes C, Helfrich-Forster C, Hall JC, Stanewsky R (2003). Curr Biol.

[61] Nitabach MN, Taghert PH (2008). Curr Biol.

[62] Masri S, Zocchi L, Katada S, Mora E, Sassone-Corsi P (2012). Ann N Y Acad Sci.

[63] Edgar RS, Green EW, Zhao Y, van Ooijen G, Olmedo M, Qin X, Xu Y, Pan M, Valekunja UK, Feeney KA, Maywood ES, Hastings MH, Baliga NS, Merrow M, Millar AJ, Johnson CH, Kyriacou CP, O’Neill JS, Reddy AB (2012). Nature.

[64] Salomé PA, Oliva M, Weigel D, Krämer U (2013) EMBO J 32:511–523 10.1038/emboj.2012.330 PMC3579136 23241948

[65] Hong S, Kim SA, Guerinot ML, McClung CR (2013) Plant Physiol 161:893–903 10.1104/pp.112.208603 PMC3561027 23250624

[66] Chen YY, Wang Y, Shin LJ, Wu JF, Shanmugam V, Tsednee M, Lo JC, Chen CC, Wu SH, Yeh KC (2013) Plant Physiol [Epub ahead of print] 10.1104/pp.112.212068 PMC3585605 23307650

[67] Metzendorf C, Wu W, Lind MI (2009). Biochem J.

